# Quasi-experimental pilot study to improve mobility and balance in recurrently falling nursing home residents by voluntary non-targeted side-stepping exercise intervention

**DOI:** 10.1186/s12877-022-03696-y

**Published:** 2022-12-30

**Authors:** Frédéric Dierick, Anne-France Bouché, Serge Guérin, Jean-Paul Steinmetz, Carine Federspiel, Vincent Barvaux, Fabien Buisseret

**Affiliations:** 1 Centre National de Rééducation Fonctionnelle et de Réadaptation – Rehazenter, Laboratoire d’Analyse du Mouvement et de la Posture (LAMP), Rue André Vésale 1, 2674 Luxembourg, Luxembourg; 2 “Le Richemont”, Physiotherapy Department, Korian Group, Rue de L’Enclos 13, 5537 Bioul-Anhée, Belgium; 3 INSEEC Grande Ecole, Avenue Claude Vellefaux 27, 75010 Paris, France; 4 Zitha, Rue Burgkapp 4-5, 6211 Consdorf, Luxembourg; 5 Zitha, Rue Ste Zithe 30, 2763 Luxembourg, Luxembourg; 6 Haute Ecole Louvain en Hainaut, Rue de l’Hôpital 2, 6060 Gilly, Belgium; 7 CeREF-Technique, Chaussée de Binche 159, 7000 Mons, Belgium

**Keywords:** Falls, Prevention, Walking, Exercise, Training, Rehabilitation

## Abstract

**Background:**

Side-stepping is a potential exercise program to reduce fall risk in community-dwelling adults in their seventies, but it has never been tested in nursing home residents. This was a pilot quasi-experimental study to examine the feasibility and potential mobility and balance benefits of an intervention based on voluntary non-targeted side-stepping exercises in nursing home residents who fall recurrently.

**Methods:**

Twenty-two participants were recruited and non-randomly assigned to an intervention group ($n=$11, side-stepping exercises, STEP) participating in an 8-week protocol and to a control group ($n =$11, usual physiotherapy care, CTRL). They were clinically assessed at 4-time points: baseline, after 4 and 8 weeks, and after a 4-week follow-up period (usual physiotherapy care). Statistical differences between time points were assessed with a Friedman repeated measures ANOVA on ranks or a one-way repeated measures ANOVA.

**Results:**

Compared to baseline, significant benefits were observed in the STEP group at 8 weeks for the Timed Up and Go ($p =$0.020) and 6-minute walking test ($p =$0.001) as well as for the Berg Balance Scale ($p =$0.041) and Mini motor test ($p =$0.026). At follow-up, the Tinetti Performance Oriented Mobility Assessment and Berg Balance Scale significantly worsened in the STEP group ($p =$0.009 and $p<$0.001, respectively). No significant differences were found between the groups at the same time points.

**Conclusions:**

Our intervention was feasible and improved mobility and balance after almost 8 weeks. Studies with larger samples and randomized control trials are needed to consolidate our preliminary observations and confirm the deterioration of some tests when side-stepping exercises are discontinued.

**Trial registration:**

Identifier: ISRCTN13584053. Retrospectively registered 01/09/2022.

## Background

Falls in older adults are a major public health issue in all countries. Despite professional supervision, the institutionalization of older adults in nursing homes cannot eliminate falls, fall-related injuries, and subsequent disabilities. Fortunately, a proven strategy to prevent falls in older adults is to implement exercise interventions [1, 2]. Therefore, it is of utmost importance to investigate the feasibility, and mobility and balance benefits of implementing new exercise protocols into the daily routine of nursing home residents, specifically those who experience recurrent falls.

Both reactive and voluntary stepping exercises are efficient in reducing fall rates by approximately 50% in older adults, in both community and nursing home settings [3], and therefore should be used and recommended for nursing home residents. To the best of our knowledge, previous studies of voluntary stepping exercises in older adults have mostly used technology-based targets or distractors, materialized by mats regularly partitioned into squares (25cm$\times$25cm) [4–8], mats with colored squares (10cm$\times$10cm) [9], exercise-based video gaming (exergames) pads with buttons [10, 11], pressure/force sensitive platforms [12, 13] or a camera [14]. A study using colored lines taped to the floor was also conducted [15], but the visual demand of such a task is high.

Although the use of targets/ distractors in this context has been shown to be effective for improving mobility and balance in older adults, these protocols preferentially involve the processing of visual information and additional cognitive stimuli [3] to accurately place the foot on the expected target and avoid distractors rather than involving proprioceptive information. Here, we developed a new protocol for training voluntary side-stepping in older adults that does not require the use of targets or distractors and requires a few inexpensive devices. Our protocol proposes to focus mainly on the proprioceptive training of the hip muscles and joint structures during the sideways displacement and positioning of the foot. Moreover, such a non-targeted side-stepping protocol might be easier to implement in nursing home residents with lower cognitive abilities.

Our hypothesis is that training based solely on voluntary non-targeted side-stepping exercises might be feasible and could improve the mobility and balance of nursing home residents who fall recurrently. Indeed, voluntary sideways steps is a more active displacement because it requires more central nervous system involvement, in contrast to rhythmic forward walking, which is mainly passive and relies on the spontaneous pendular dynamics of lower limbs [16]. Voluntary sideways steps could also specifically target the strengthening of the frontal plane hip muscles (abductors/ adductors) which play an essential role in stabilizing the head, arms, and trunk in the frontal plane during standing and walking [17]. Moreover, the feasibility and benefits of a 6-week non-targeted sideways steps intervention in a small group of community-dwelling adults in their seventies have already been demonstrated [18]. In this last study, the proposed way to increase the level of difficulty for participants during the successive sessions of the intervention was to increase their self-selected walking pace, if they were able to do so. This modality is difficult to implement for nursing home residents with limited cognitive abilities. Here, six progressively increasing levels of difficulty, allowing personalized training for each resident, were developed.

The aim of this study is to examine the feasibility and potential benefits of a standardized, non-targeted, voluntary side-stepping intervention over an 8-week period on the mobility and balance of a small group of nursing home residents who fall recurrently compared with a non-randomized control group who completed their usual physiotherapy program. Outcomes were assessed in both groups using conventional clinical tests before the intervention, after 4 and 8 weeks of the intervention, and after a 4-week follow-up.

## Methods

### Participants

All participants were recruited from the nursing home “Le Richemont” (Bioul, Belgium), with a total of 45 nursing and care home residents. The experimental protocol was performed in line with the principles of the Declaration of Helsinki. All methods were carried out in accordance with relevant guidelines and regulations. Ethical approval for all procedures was granted by the Academic Bioethics Committee (B200-2017-090). Informed consent was obtained from each participant or legal guardian.

The inclusion and exclusion criteria were as follows. Each participant had to be over 60 years old, be a recurrent faller, be able to stand and walk alone or with technical/ verbal assistance for a distance of 10 meters, and be able to understand the instructions given for intervention and assessment. A recurrent faller was defined as an individual who had fallen two or more times within a specified time period [19], in this case, the past 12 months. Participants with severe vascular disease or epileptic seizures were excluded.

### Study design and data collection procedures

The pilot study design was a quasi-experimental controlled trial, without random assignment. Thirty-five participants were screened for eligibility and 22 were recruited to participate in the study. Eleven participants were assigned to an intervention group (side-stepping exercises, STEP) and eleven to a control group (usual physiotherapy care, CTRL). The characteristics of the participants are shown in Table 1.

**Table 1:** Participant’s characteristics, baseline (t1) assessment for usual care (CTRL) and side-stepping intervention (STEP) groups, and co-morbidities. Results are expressed as counts, mean±SD or median[q1–q3]. Walking aid (no (n) or yes (y)), aid type (crutch (c), walker (w), or verbal (v)), Elderly Mobility Scale (EMS, scored /20), Falls Risk Assessment Tool (FRAT, scored /20), Get-up early test (GUE, scored /4), Stops walking when talking (SWWT, no (n) or yes (y)). Katz category B: Physical dependent for bathing, dressing, transferring and/or toileting. Mental dependent, disoriented in time and space and dependent for bathing and/or dressing. Katz category Cd: Mental dependent, disoriented in time and space and diagnosed for dementia by a specialist physician. Physical dependent for bathing, dressing, transferring and/or toileting and/or feeding and incontinent

	Usual care (CTRL) *n*=11	Side-stepping intervention (STEP) *n*=11	t/U/$\bf \chi ^2$	*p*
Sex (F/M)	10F/1M	9F/2M	$\chi ^2$=0.749	0.387
Age (years)	84±7	86±8	t=-0.652	0.522
Walking aid (y/n)	8y/3n	8y/3n	$\chi ^2$=0.234	0.629
Aid type (c,w,v)	6w/2v	1c/6w/2v	$\chi ^2$=9.472	0.149
Katz (A-D)	10B/1Cd	7B/4Cd	$\chi ^2$=0.088	0.766
EMS (/20)	13[9–14]	15[7–15]	U=42.0	0.233
FRAT (/20)	15[12–17]	13[11–17]	U=53.0	0.644
GUE (/4)	1[1–2]	0[0–2]	U=145.0	0.215
SWWT (y/n)	9y/2n	9y/2n	$\chi ^2$=0.076	0.782
TUG (s)	46.2±22	52.6±31	t=0.554	0.586
6MWT (m)	112.5±56	105.4±88	t=-0.225	0.824
6mWT (s)	18.3±7	18.9±11	t=0.143	0.888
Tinetti (/28)	18[16–25]	20[18–21]	U=52.5	0.618
BBS (/56)	28[20–32]	29[21–32]	U=60.0	1.000
MMT (/20)	11[9–12]	11[9–14]	U=56.5	0.816
MMSE<24 (N)	3	8		
High blood pressure* (N)	2	8		
Depression (N)	2	4		
Dizziness (N)	1	0		
Hypothyroidism (N)	1	2		
Hypercholesterolemia (N)	2	2		
Schizophrenia (N)	0	1		
Heart disease (N)	1	1		
Cancers (N)	2	1		
Asthma (N)	1	0		
Anemia (N)	0	1		
Deafness (N)	0	1		
Cardiac arrhythmia (N)	0	1		
Insomnia (N)	0	1		
Herpes infection (N)	0	1		
Visual impairment (N)	1	1		

t-test (t) for age, TUG, 6MWT, and 6mWT; Mann-Whitney rank sum test (U) for EMS, FRAT, GUE, Tinetti, BBS, and MMT; Chi square ($\chi ^2$) for sex, walking aid, aid type, Katz, and SWWT. MMSE: Mini-Mental State Examination [50], * value>140/90 mmHg

Participants in the STEP and CTRL groups were examined in the morning, at 4-time points (t1–t4): at baseline, within 2–3 days before the start of side-stepping exercises/usual physiotherapy care (t1), after 4 weeks (t2) and 8 weeks (t3) of side-stepping exercises/usual care, and at follow-up, 4 weeks after the intervention/usual care (t4). Note that usual physiotherapy care was provided between t3–t4 in both groups. Falls were defined as “an unexpected event in which the participant comes to rest on the ground, floor, or at a lower level” [20] and were recorded during the intervention/usual care and follow-up periods. This definition was agreed upon with all nursing home staff and adopted for recordkeeping. It excludes trips, which do not result in a participant coming to rest on the ground/floor/lower level because he/she successfully regains balance. In the nursing home, falls for all residents are systematically recorded by the staff member responsible for counting falls, and reports are written and included in the residents’ fall care plans and in a paper-based falls diary for all residents.

Sex, age, walking aids, and co-morbidities were recorded for all participants (Table 1). Katz scale [21], Elderly Mobility Scale (EMS) [22], Falls Risk Assessment Tool (FRAT) [23], Get-up early test (GUE) [24], and Stops walking when talking (SWWT) [25] were collected at baseline to complete the description of participants in the STEP and CTRL groups (Table 1). A three-level fall risk using the FRAT score was used to categorize the participants: low (5–11), medium (12–15), and high (16–20) [23].

### Outcome measures

Several other clinical tests were assessed from baseline to the end of follow-up (t1–t4): Berg Balance Scale (BBS) [26], Tinetti Performance Oriented Mobility Assessment (Tinetti) [27], Mini motor test (MMT) [28], 6-minute walking test (6MWT) [29], 6-meter walking test (6mWT) [30], and Timed Up and Go (TUG) [31].

All of these tests were administered by the same experienced physiotherapist, who did not participate in the study and was blinded to group assignment (she worked afternoons and was instructed not to ask participants whether or not they took side-stepping exercises). All tests were performed by participants wearing the same orthopedic shoes they wore at baseline, during the intervention, and at follow-up.

### Intervention

The intervention was always performed in the morning and consisted of replacing the usual physiotherapy care sessions (total duration of about 120 minutes per week: 20–25 minutes per day) with an intervention consisting solely of voluntary side-stepping exercises. In the nursing home, the usual physiotherapy sessions mainly included walking on level ground, ascending and descending stairs, and upper and lower limb strengthening exercises.

During the side-stepping exercises intervention period (t1–t3), participants were trained 4 days per week for 30 minutes each. Daily training time could be divided into two periods of 15 minutes if the participant had difficulty participating in a single 30-minute session because of fatigue or other reasons.

**Fig. 1:**
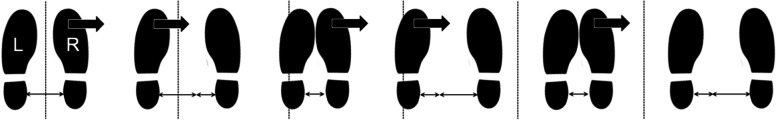
Description of right-hand side-stepping movements. L: left foot, R: right foot. Note that for safety reasons the feet never cross each other

**Fig. 2:**
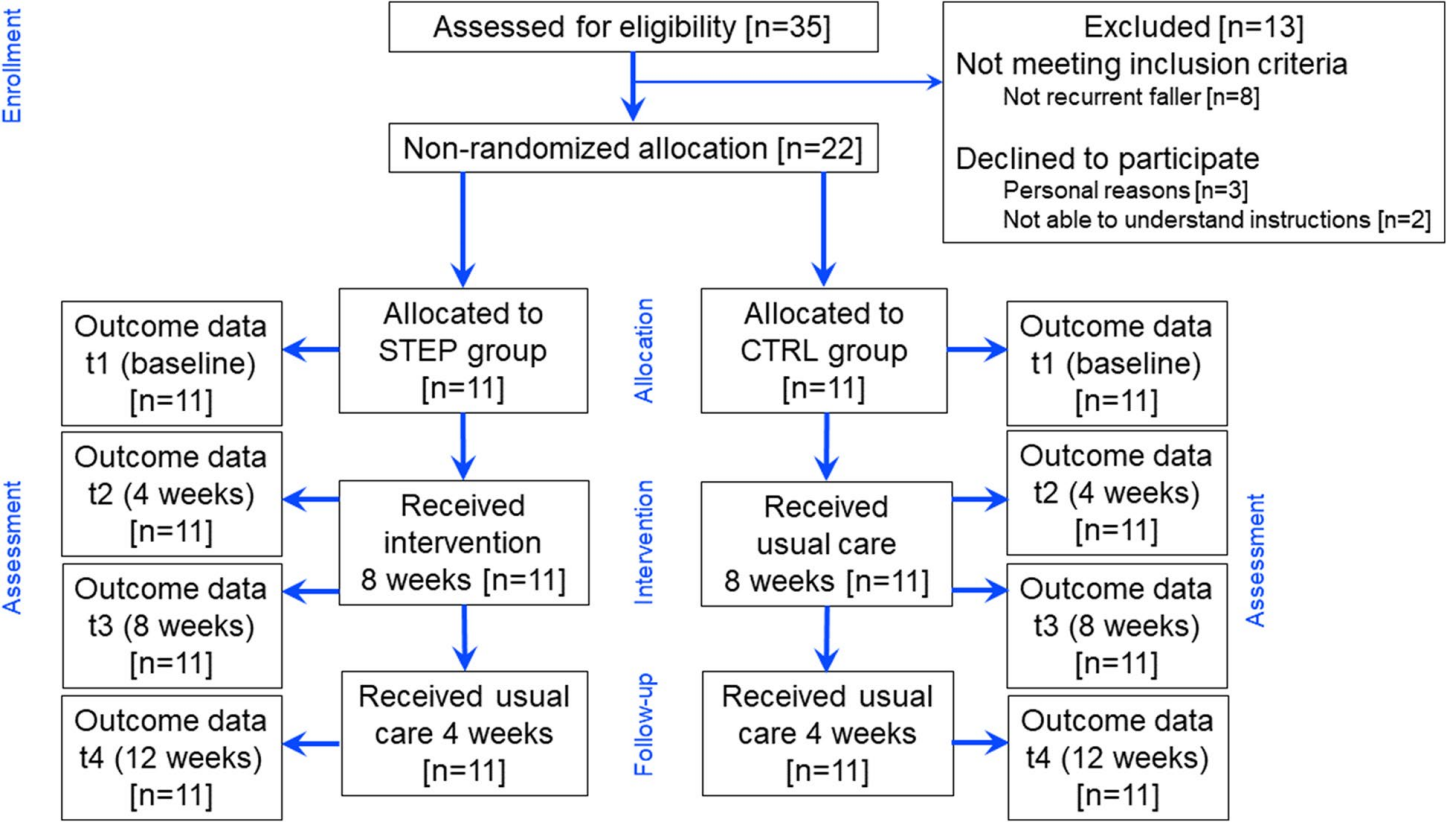
Flow diagram of the enrollment, allocation, intervention, and assessment processes. Note that the study design was quasi-experimental and allocation in the STEP and CTRL groups was not randomized

Voluntary side-stepping movements were performed with the same orthopedic shoes used in the assessment tests, in left and right directions, in front of a horizontal bar located 90cm from the floor and situated in a corridor. For the right-hand side-stepping movements, the right foot was moved approximately 15–20cm to the right, then the left foot to join the right, and so on (Fig. 1). For the left-hand side-stepping movements, the reverse order was chosen. Participants were instructed to perform the sideways steps at a frequency of 1s$^{-1}$ ($\approx$1500 steps session$^{-1}$) while keeping their heads in a neutral position, looking straight ahead, and watching the position of their feet as little as possible. For safety reasons, participants were asked never to cross their feet (Fig. 1). The intervention process is shown in Fig. 2.

Since the participants in the STEP group did not have the same level of mobility and balance abilities at baseline (see results at t1 in Table 2), six levels of difficulty were established for the side-stepping exercises, allowing progression for each of them. For *level 1*: side-stepping movements were performed on the floor, with both hands maximally supported on the bar. For *level 2*: side-stepping movements were performed on two 1.5cm thick exercise mats with a total length of 400cm (width$\times$length: 100$\times$200cm, Corona, Airex, Sins, Switzerland), and two latex sleep mattresses of 14cm thick on a total length of 400cm (width$\times$length: 90$\times$200cm), with a maximum of both hands supported on the bar. For *level 3*: side-stepping movements were performed on the floor, both index fingers resting lightly on the bar. For *level 4*: side-stepping movements were performed on mats and mattresses, with both index fingers resting lightly on the bar. For *level 5*: side-stepping movements were performed on the floor, without support from hands/fingers. Finally, for *level 6*: side-stepping movements were performed on the mats and mattresses, without hands/fingers support. All participants started at *level 1* and the decision whether to move to the next level or a thicker mat was left to the visual assessment of the physiotherapist (A.-F.B.), who assessed the quality of balance, speed of execution of the sideways steps, fluidity of lower limb movements, and endurance of the participants.

**Table 2:** Friedman RM ANOVA on ranks (Q) or one-way RM ANOVA (F) results for clinical tests at the 4-time points (t1–t4) for the intervention (STEP) and usual physiotherapy care (CTRL) groups. Significant results are in bold and significant multiple comparisons are also presented. The following results are given: Berg Balance Scale (BBS), Tinetti Performance Oriented Mobility Assessment (Tinetti), Mini motor test (MMT), 6-minute walking test (6MWT), 6-meter walking test (6mWT), and Timed Up and Go (TUG). Effect sizes are reported in the last column as Cohen’s *f* or Kendall’s *W*

	t1 (baseline)	t2 (4 weeks)	t3 (8weeks)	t4 (follow-up)	F(*p*) or Q(*p*)	Significant post-hoc (*p*)	Effect size *f* or *W*
Intervention (STEP) *n* = 11							
TUG (s)	52.6±31	42.7±23	34.9±22	26.8±23	F=7.583(**<0.001**)	t1–t4 (**<0.001**), t1–t3 (**0.020**), t2–t4 (**0.034**)	*f*=0.87 (large)
6MWT (m)	105.4±88	137.0±83	157.7±111	134.9±87	F=6.255(**0.002**)	t1–t3 (**0.001**)	*f*=0.79 (large)
6mWT (s)	13.7[11.2–25.0]	14.0[9.6–24.0]	16.3[11.3–21.0]	18.1[12.0–22.9]	Q=0.491(0.921)	–	*W*=0.01 (negligible)
Tinetti (/28)	20[18–21]	19[18–24]	21[16–24]	17[11–20]	Q=13.690(**0.003**)	t3–t4 (**0.009**)	*W*=0.41 (large)
BBS (/56)	29[21–32]	28[19–31]	33[22–39]	21[14–28]	Q=20.611(**<0.001**)	t3–t4 (**<0.001**), t2–t3 (**0.005**), t1–t3 (**0.041**)	*W*=0.62 (large)
MMT (/20)	11[9–14]	12[11–13]	15[11–16]	13[10–16]	Q=12.949(**0.005**)	t2–t3 (**0.012**), t1–t3 (**0.026**)	*W*=0.39 (moderate)
Usual physiotherapy care (CTRL) *n*=11							
TUG (s)	46.2±22	47.7±19	46.5±19	47.1±15	F=0.200(0.896)	–	*f*=0.14 (small)
6MWT (m)	112.6±56	115.8±38	115.7±54	114.8±44	F=0.152(0.928)	–	*f*=0.12 (small)
6mWT (s)	15.1[12.1–26.5]	16.9[12.7–24.5]	19.3[16.2–24.5]	18.1[14.9–26.5]	Q=7.691(0.053)	–	*W*=0.23 (small)
Tinetti (/28)	18[16–25]	19[18–24]	19[16–23]	19[15–24]	Q=6.000(0.112)	–	*W*=0.18 (small)
BBS (/56)	28[20–32]	29[22–31]	29[20–33]	28[21–33]	Q=3.487(0.322)	–	*W*=0.10 (small)
MMT (/20)	11[9–12]	10[9–12]	11[9–13]	11[9–13]	Q=3.792(0.285)	–	*W*=0.11 (small)

Significant *p*-values are in bold

### Sample size estimation and statistical analysis

A priori estimation of the sample size was done with G*Power software (version 3.1.9.7), with an $\alpha$ level (I) of 0.05 and $\beta$ level (II) of 0.20, and with a statistical power of 0.80. The estimation was made on the mean results of [18], who reported a significant TUG time decrease (10.16±1.51 versus 8.79±1.72s, $p<$0.001, Cohen’s *f*=0.95 – large effect size) after a 6-week side-stepping intervention in community-dwelling adults in their seventies. An effect size *dz* of 0.94 was calculated for the bilateral t-test for paired samples and a correlation between groups of 0.6. The total estimated sample size was 11.

Data were tested for normality (Shapiro-Wilk) and tests for equal variance (Brown-Forsythe) were performed. BBS, Tinetti, and MMT results were tested with a Friedman repeated measures ANOVA on ranks (Friedman’s *Q*) for factor *time* (t1–t4). *Post-hoc* analyses were performed with Tukey’s test to ensure multiple comparisons of the values obtained over t1–t4. Data from 6MWT, 6mWT, and TUG were analyzed with a one-way repeated measures ANOVA (RM ANOVA) for factor *time* or Friedman repeated measures ANOVA on ranks when the normality test failed. *Post-hoc* analyses were performed using the Holm-Sidak method to ensure multiple comparisons of the values obtained over t1–t4.

For one-way RM ANOVA results, effect sizes were calculated using Cohen’s *f* and interpreted as follows: $f<$0.10 negligible, 0.10$\le f<$0.25 small, 0.25$\le f<$0.40 moderate, and ${f}\ge$0.40 large. For Friedman’s *Q* results, effect sizes were calculated using Kendall’s coefficient of concordance *W* with an identical interpretation to that of Cohen’s *f*.

Since our goal was to study the impact of side-stepping on mobility and balance versus time in STEP and CTRL groups (within-group comparison), we do not systematically compare STEP and CTRL groups at the same time points (between-group comparison). Within-group comparison design increases the chance of discovering a real difference among the different time periods, by minimizing the random noise linked to individual factors of each resident that cannot all be controlled. Mann-Whitney rank sum tests or two-way RM ANOVA tests (*group*, *time*, *group*$\times$*time*) with *post-hoc* Holm-Sidak method may however be performed if some peculiar outcomes show changes beyond the minimal detectable change at 95% (MDC$_{95}$) in similar populations for the different clinical tests: 5.37s for TUG [32], 50m for 6MWT [33], 15.2s for 6mWT [34], 4.0–4.2 points for Tinetti [35], 7–8 points for BBS [34, 36], and the MDC$_{95}$ value for MMT is unknown to our knowledge.

The significance threshold for all statistical tests was set at $\alpha =$0.05. Data analysis was performed with Sigmaplot (v.11.0, Systat Software, San Jose, CA) and R software (v. 4.2.1, R Foundation for Statistical Computing, Vienna, Austria) [37].

## Results

The participants’ path through the study is shown in Fig. 2. There were no losses at follow-up. Of the 11 participants recruited in the STEP group, all were able to successfully complete the 8-week intervention. Only 3 participants were able to perform side-stepping sessions of 30 minutes at a time. At the end of the intervention, 8 participants reached *level 3* and 3 participants reached *level 4* (with mattresses thickness of 10cm). One participant in the STEP group fell during t2–t3, but outside the intervention and without consequences for further participation in the study. Two participants in the STEP group reported only mild discomfort related to muscle soreness or stiffness during t1–t2.

Participants’ characteristics and clinical test results at t1 (baseline) are listed in Table 1. No significant differences were found between the groups. In particular, fall risk was nearly identical between the CTRL (low: *n*=2 participants, medium: *n*=4, and high: *n*=5) and STEP (low: *n*=4 participants, medium: *n*=5, and high: *n*=2) groups.

Clinical test results at the different time points are shown in Table 2 for the two groups. In the STEP group, during t1–t3, mean TUG decreased significantly by 17.7s (*p*=0.020), 6MWT value increased by 52.3m (*p*=0.001), and median BBS (*p*=0.041) and MMT scores (*p*=0.026) increased by 4 points (Table 2). During t2–t3, median BBS (*p*=0.005) and MMT scores (*p*=0.012) increased significantly by 5 and 3 points, respectively (Table 2). During t3–t4, median Tinetti (*p*=0.009) and BBS scores ($p<$0.001) decreased significantly by 4 and 12 points, respectively (Table 2). Effect sizes for these clinical variables were negligible to large in the STEP group (Table 2). Still in the STEP group, no significant differences were observed for 6mWT at the different time points. No significant differences in clinical tests were observed at any time point in the CTRL group (Table 2). Effect sizes were small for all clinical variables in the CTRL group (Table 2).

No significant differences were found between the groups at the same time points. Only non-significant differences in TUG at t3 and t4 were beyond the MDC$_{95}$ (t3=11.7s, t=1.142, *p*=0.274 and t4=20.3s, t=1.986, *p*=0.068).

## Discussion

The aim of this study was to evaluate the feasibility and potential benefits of an 8-week of solely voluntary side-stepping exercise intervention ($\approx$1500 steps session$^{-1}$ at a frequency of 4 sessions week$^{-1}$, i.e. a total of $\approx$48000 steps) on TUG, 6MWT, 6mWT, Tinetti, BBS, and MMT in nursing home residents who fall recurrently. A standardized side-stepping intervention with increasing levels of difficulty was developed. In the literature, a stepping intervention is defined as “training of single or multiple volitional or reactive steps in an upright position in response to an environmental challenge” [3]. Our intervention fits this definition because the voluntary sideways steps are performed in an upright position on unstable surfaces with two different thicknesses.

Since many previous trials included in systematic reviews and meta-analyses [1, 2, 38] examining the effects of exercises on mobility and balance abilities in older adults were multimodal (stepping, balance, strengthening, resistance training, ...), understanding the isolated effects of a stepping intervention in a particular direction of movement on mobility and balance abilities is still of great clinical importance for fall prevention. Here, we chose to investigate the benefits of voluntary sideways steps. To our knowledge, this is the first study to investigate this modality in nursing home residents who fall recurrently and therefore complements in a beneficial way a recent clinical trial that evaluated the effectiveness of a side-stepping intervention in community-dwelling fallers and non-fallers adults in their seventies [18]. We were guided in our choice by the results of a systematic review and meta-analysis showing that voluntary step training may have greater effects on fall risk than reactive step training, and by the assumption that voluntary step training may prevent falls that require pre-planned altered step patterns and adaptability of gait [3]. However, in this last reference, studies of voluntary step training have longer training times, higher doses, and additional cognitive stimulation compared to studies of reactive step training. Further standardized studies comparing these voluntary and reactive stepping modalities in different directions are needed to determine the most efficient protocols.

At baseline, the nursing home residents participating in the study had major deficits in mobility and balance, which could be explained mainly by their very advanced age, their various co-morbidities, and the use of walking aids by more than half of them (mainly walkers). The TUG time was 53±31s in the STEP group and 46±22s in the CTRL group. These values are higher than those observed in similarly aged adults living in residential care facilities (28±15 to 30±17s) [39]. However, values between 10 and 109s were observed in this last study, and the walking aids used by the residents were not reported. It is important to note that longer TUG times were reported when a cane [40] or a walker [41] was used. The 6MWT distance was 105.4±88m in the STEP group and 112.6±56m in the CTRL group, corresponding to a self-selected gait speed of $\approx$ 0.30m s$^{-1}$, and the median 6mWT time was 13.7s in the STEP group and 15.1s in the CTRL group, corresponding to a speed of $\approx$ 0.40m s$^{-1}$. Our gait speed results are slightly lower than or compatible with the speed of similarly aged adults living in residential care facilities (0.4±0.2m s$^{-1}$) [39] and the definition of household ambulators (<0.40m s$^{-1}$) [42]. The Tinetti score was 20[18–21] points in the STEP group and 18[16–25] in the CTRL group. These scores are also consistent with the results of a study conducted on old adult nursing home residents (20 points) [43]. The BBS score was 29[21–32] points in the STEP group and 28[20–32] in the CTRL group. These scores also correspond to the ranges of 8 to 55 and 3 to 54 points observed in two previous studies [36, 39]. The MMT score was 11[9–14] in the STEP group and 11[9–12] in the CTRL group, which are also consistent with the results of a study in patients with psychomotor disadaptation syndrome (13±5 points) [28]. Overall, our baseline results show mobility and balance abilities comparable to those previously observed in similar groups.

Inspection of the clinical results shows that the minimum (maximum) clinical test values were observed at t3, followed by an increase (decrease) at t4. Thus, participants tend to reach optimal performance immediately after the training period, which then tends to decline during the follow-up. This observation was true for all clinical variables assessed, except for TUG which shows a quasi-perfect linear decrease between t1–t4. During this period, TUG was improved by half the time in the STEP group but this result could not be explained by a decrease in gait phases duration since 6mWT was statistically unchanged and duration was even increased by 5s (Table 2), with a negligible effect size. In nursing home residents with a TUG above 30.01 seconds, which is the case of the residents included in our study, researchers observed that 50% of the time spent to realize the test is related to mid- and end-half-turning phases [44]. More, in elderly adults (69 to 92 years) who had difficulty in turning, these half-turning phases are mainly characterized by multiple steps or weight shifts instead of the full-pivots type of turn, i.e. body rotations observed in young adults [45]. These multiple steps or weight shifts have a predominantly side component which was specifically worked on during our intervention. Therefore, we believe that the decreased time of TUG in STEP group is directly related to the decreased time during the half-turning phases, probably explained by a reduced number of steps. This hypothesis must be tested in a future study.

Our intervention results showed significant benefits after 8 weeks of side-stepping exercises for TUG, 6MWT, BBS, and MMT. Note that a period of 4 weeks (t2) was too short to show significant differences. Therefore, we recommend planning a side-stepping intervention of almost 8 weeks. On the other hand, the follow-up period with classical physiotherapy care without sideways steps showed a significant worsening of Tinetti and BBS. However, a statistically significant difference does not mean that there is a true improvement/deterioration in the mobility and balance abilities of the participants. Therefore, our results were supplemented by calculating the effect sizes that showed moderate to large effects. Our results should be discussed with the values of the MDC$_{95}$ and we can conclude that the changes in TUG (–17.7s) and in 6MWT (+52.3m) values can be considered as actual improvements in functional mobility, dynamic balance, and walking endurance. In contrast, the change observed for BBS (+4 points) score over the same period is not sufficient to conclude a true improvement in static balance ability. Note that our intervention is dynamic in nature and the absence of improvement in static balance is not therefore surprising.

After the follow-up period, the changes in Tinetti (–4 points) and BBS (–12 points) scores are large enough to infer a true deterioration in balance abilities when participants no longer perform the side-stepping exercises. This result should be considered when implementing a protocol with only side-stepping exercises in nursing home residents who fall recurrently because the risk of falling might increase when residents stop exercising. Fortunately, despite a deterioration in balance abilities after follow-up, no falls were reported here during this period. Given the current state of knowledge, we recommend that sideways steps not be performed solely in daily physiotherapy practice in nursing homes, but definitely in combination with more classical physiotherapy exercises to limit this potentially harmful effect, for example, if intensive sideways steps training must be abruptly terminated (hospitalization, sudden deterioration of general health, ...).

The estimated sample size for each group was 11 residents. We decided to create two very small groups to minimize the financial impact of the study. We, therefore, opted for a detailed intra-group comparison rather than a comparison between groups, which would have introduced much more random noise explained by factors (history, knowledge, context, ...) that cannot all be controlled for in this design. In addition to our very small groups, which were estimated a priori based on measurements done in younger participants living in the community [18], several limitations of this study should be mentioned. The strength of frontal plane hip muscles was not studied. However, a decrease in the maximum strength of the hip abductor/ adductor muscles in older adults leads to deficits in static and dynamic balance [46]. Interpretation of MMT results was not possible because no value for MDC$_{95}$ is reported in the literature. Comparison with a group of nursing home residents who did not fall might have shown a different benefit of the intervention in terms of mobility and balance abilities. We performed only a single-task TUG that may have overestimated the functional mobility capacity of our participants. We could also have obtained results with a cognitive dual-task TUG [47]. Finally, the instrumentation of several clinical tests selected in this study with low-cost, wearable inertial sensors is easy to implement in elderly nursing home residents and could have provided a more quantitative and informative assessment of fall risk [48, 49]. However, even today, these instrumented tests are largely reserved for motion scientists with the expertise to analyze the recorded time series. They also require a considerable amount of additional time to perform the tests and, more importantly, to analyze the data, which is difficult to reconcile with a daily physiotherapy practice in nursing homes.

## Conclusions

Our protocol with only side-stepping exercises with different levels of difficulty allowing progressive and personalized training, performed over almost an 8-week period, was feasible and improve the mobility and balance of nursing home residents who fall recurrently. These preliminary results suggest that voluntary side-stepping exercises may be an appropriate intervention for nursing home residents in the hope of reducing the frequency and severity of falls. However, the deterioration in balance abilities observed at follow-up is of clinical relevance and suggests that side-stepping exercises should not be abruptly terminated in older adults. Further studies with larger samples and randomized control trials are needed to consolidate our preliminary but promising observations.

## Data Availability

The datasets analyzed during the current study are available from the corresponding author.
